# 4-[(4-Chloro­phen­yl)(5-hydr­oxy-3-methyl-1-phenyl-1*H*-pyrazol-4-yl)meth­yl]-5-methyl-2-phenyl-1*H*-pyrazol-3(2*H*)-one

**DOI:** 10.1107/S1600536808036088

**Published:** 2008-11-08

**Authors:** Hoong-Kun Fun, Samuel Robinson Jebas, K. S. Girish, B. Kalluraya

**Affiliations:** a X-ray Crystallography Unit, School of Physics, Universiti Sains Malaysia, 11800 USM, Penang, Malaysia; b Department of Studies in Chemistry, Mangalore University, Mangalagangotri, Mangalore 574199, India

## Abstract

In the the title compound, C_27_H_23_ClN_4_O_2_, the chloro­phenyl ring forms dihedral angles of 77.70 (9) and 86.65 (9)°, respectively, with the pyrazol-3-one and pyrazole rings. The phenyl rings attached to the pyrazole rings are twisted away from them [dihedral angles 33.80 (9) and 40.34 (10)°]. An intramolecular O—H⋯O hydrogen bond generates an *S*(8) ring motif. The mol­ecules are linked into chains running along the *c* axis by N—H⋯N hydrogen bonds, and the chains are cross-linked *via* C—H⋯O hydrogen bonds and C—H⋯π inter­actions involving the chloro­phenyl ring.

## Related literature

For the biological activities of pyrazoles, see: Burger & Iorio (1979 ▶); Holla *et al.* (1994 ▶); Kalluraya & Chimbalkar (2001 ▶); Windholz (1976 ▶); Wolff (1980 ▶). For bond-length data, see: Allen *et al.* (1987 ▶). For hydrogen-bond motifs, see: Bernstein *et al.* (1995 ▶).
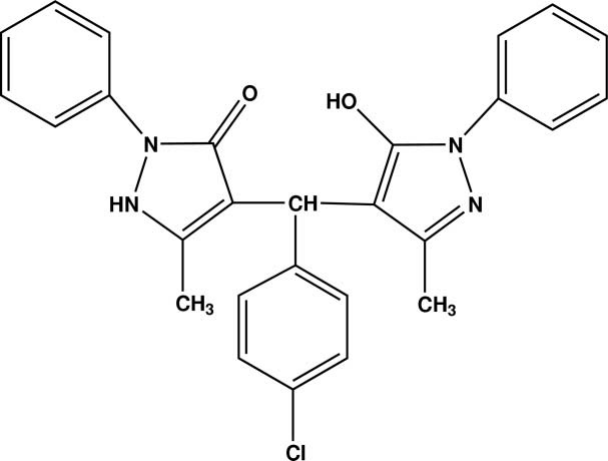


## Experimental

### 

#### Crystal data

C_27_H_23_ClN_4_O_2_
*M* *_r_* = 470.94
Monoclinic, 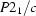

*a* = 10.8809 (2) Å
*b* = 11.2046 (2) Å
*c* = 18.9376 (3) Å
β = 97.994 (1)°
*V* = 2286.36 (7) Å^3^
*Z* = 4
Mo *K*α radiation
μ = 0.20 mm^−1^
*T* = 100.0 (1) K
0.41 × 0.13 × 0.07 mm

#### Data collection

Bruker SMART APEXII CCD area-detector diffractometer
Absorption correction: multi-scan (*SADABS*; Bruker, 2005 ▶) *T*_min_ = 0.923, *T*_max_ = 0.987
26858 measured reflections
6751 independent reflections
4614 reflections with *I* > 2σ(*I*)
*R*_int_ = 0.072

#### Refinement

*R*[*F* ^2^ > 2σ(*F* ^2^)] = 0.054
*wR*(*F* ^2^) = 0.121
*S* = 1.02
6751 reflections
317 parameters
2 restraints
H atoms treated by a mixture of independent and constrained refinement
Δρ_max_ = 0.46 e Å^−3^
Δρ_min_ = −0.36 e Å^−3^

### 

Data collection: *APEX2* (Bruker, 2005 ▶); cell refinement: *SAINT* (Bruker, 2005 ▶); data reduction: *SAINT*; program(s) used to solve structure: *SHELXTL* (Sheldrick, 2008 ▶); program(s) used to refine structure: *SHELXTL*; molecular graphics: *SHELXTL*; software used to prepare material for publication: *SHELXTL* and *PLATON* (Spek, 2003 ▶).

## Supplementary Material

Crystal structure: contains datablocks global, I. DOI: 10.1107/S1600536808036088/ci2705sup1.cif

Structure factors: contains datablocks I. DOI: 10.1107/S1600536808036088/ci2705Isup2.hkl

Additional supplementary materials:  crystallographic information; 3D view; checkCIF report

## Figures and Tables

**Table 1:** Hydrogen-bond geometry (Å, °)

*D*—H⋯*A*	*D*—H	H⋯*A*	*D*⋯*A*	*D*—H⋯*A*
O2—H1O2⋯O1	0.83 (1)	1.67 (1)	2.5025 (17)	176 (3)
N2—H1N2⋯N3^i^	0.86 (1)	1.90 (1)	2.7544 (19)	174 (2)
C13—H13*A*⋯O2^ii^	0.95	2.58	3.375 (2)	142
C4—H4*A*⋯*Cg*1^iii^	0.95	2.67	3.5088 (18)	147
C24—H24*A*⋯*Cg*1^iv^	0.95	2.86	3.745 (2)	155

Symmetry codes: (i) 
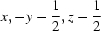
; (ii) 
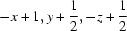
; (iii) 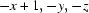; (iv) 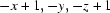. *Cg*1 is the centroid of the C11–C16 ring.

**Fig. 1.**
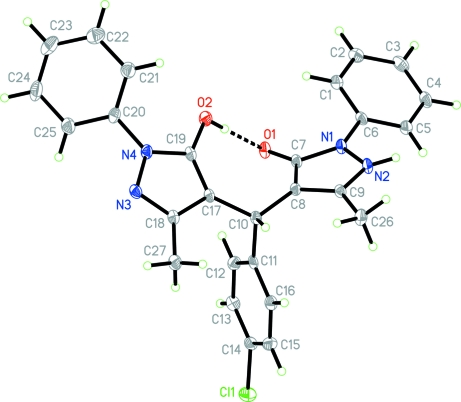
The molecular structure of the title compound, showing 50% probability displacement ellipsoids and the atom-numbering scheme.

**Fig. 2.**
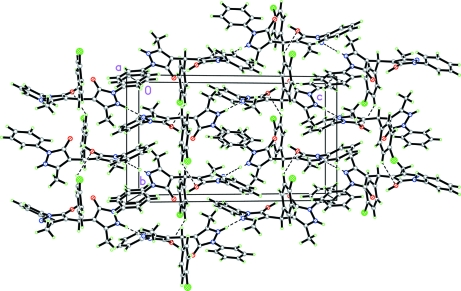
The crystal packing of the title compound, viewed along the a axis. Dashed lines indicate hydrogen bonding.

## supplementary crystallographic information

### Comment

Pyrazole derivatives have been reported to posses various biological activities such as anti-inflammatory (Windholz, 1976), analgesic, hypoglycemic (Wolff, 1980), sedative and hypnotics (Burger & Iorio, 1979), antifungal and antibacterial (Kalluraya & Chimbalkar, 2001) activities. Propenones are also found to show good antibacterial activity (Holla *et al.*, 1994). Prompted by the pharmacological applications of propenones and pyrazole derivatives, we synthesized the title compound containing the propenone-pyrazole moiety and report here its crystal structure.

Bond lengths in the title molecule (Fig.1) have normal values (Allen *et al.*, 1987). The chlorophenyl ring (C11—C16) forms dihedral angles of 77.70 (9)° and 86.65 (9)°, respectively, with the N1/N2/C7—C9 and N3/N4/C17—C19 pyrazole rings. The phenyl rings attached to the pyrazole rings are twisted; the dihedral angle between the C1—C6 and N1/N2/C7—C9 rings is 33.80 (9)° and that between C20—C25 and N3/N4/C17—C19 rings is 40.34 (10)°. An intramolecular O—H···O hydrogen bond generates an S(8) ring motif (Bernstein *et al.*, 1995).

The crystal packing shows that the molecules are linked into chains running along the *c* axis (Fig.2) by N—H···N hydrogen bonds (Table 1). The chains are cross-linked *via* C—H···O hydrogen bonds and C—H···π interactions (Table 1) involving the C11—C16 benzene ring (centroid *Cg*1).

### Experimental

The title compound was prepared by the direct fusion of 1-phenyl-3-methyl 5-pyrazolone (0.1 mole) with *p*-chlorobenzaldehyde (0.1 mole) at 413 K for 3 h. The reaction mixture was cooled to room temperature and stirred with methanol using a glass rod. The mixture was then filtered to obtain a solid product. Single crystals suitable for X-ray analysis were obtained by recrystallization from ethanol under slow evaporation (m.p. 351–353 K).

### Refinement

N– and O-bound H atoms were located in a difference map and were refined, with N—H and O—H distances restrained to 0.85 (1) Å and 0.82 (1) Å, respectively. C-bound H atoms were placed in calculated positions (C—H = 0.95–0.98 Å) and refined using a riding model with *U*_iso_(H) = 1.2*U*_eq_(C) and 1.5*U*_eq_(methyl C). A rotating group model was used for the methyl group.

### Crystal data

**Table:**

C_27_H_23_ClN_4_O_2_	*F*_000_ = 984
*M**_r_* = 470.94	*D*_x_ = 1.368 Mg m^−^^3^
Monoclinic, *P*2_1_/*c*	Mo *K*α radiation λ = 0.71073 Å
Hall symbol: -P 2ybc	Cell parameters from 3311 reflections
*a* = 10.8809 (2) Å	θ = 2.6–26.3º
*b* = 11.2046 (2) Å	µ = 0.20 mm^−^^1^
*c* = 18.9376 (3) Å	*T* = 100.0 (1) K
β = 97.994 (1)º	Plate, colourless
*V* = 2286.36 (7) Å^3^	0.41 × 0.13 × 0.07 mm
*Z* = 4	

### Data collection

**Table:**

Bruker SMART APEXII CCD area-detector diffractometer	6751 independent reflections
Radiation source: fine-focus sealed tube	4614 reflections with *I* > 2σ(*I*)
Monochromator: graphite	*R*_int_ = 0.072
*T* = 100.0(1) K	θ_max_ = 30.2º
φ and ω scans	θ_min_ = 1.9º
Absorption correction: multi-scan(SADABS; Bruker, 2005)	*h* = −15→13
*T*_min_ = 0.923, *T*_max_ = 0.987	*k* = −15→15
26858 measured reflections	*l* = −26→26

### Refinement

**Table:**

Refinement on *F*^2^	Secondary atom site location: difference Fourier map
Least-squares matrix: full	Hydrogen site location: inferred from neighbouring sites
*R*[*F*^2^ > 2σ(*F*^2^)] = 0.054	H atoms treated by a mixture of independent and constrained refinement
*wR*(*F*^2^) = 0.121	*w* = 1/[σ^2^(*F*_o_^2^) + (0.0491*P*)^2^ + 0.4127*P*] where *P* = (*F*_o_^2^ + 2*F*_c_^2^)/3
*S* = 1.02	(Δ/σ)_max_ = 0.001
6751 reflections	Δρ_max_ = 0.46 e Å^−^^3^
317 parameters	Δρ_min_ = −0.36 e Å^−^^3^
2 restraints	Extinction correction: none
Primary atom site location: structure-invariant direct methods	

### Special details

**Table:**

Experimental. The data was collected with the Oxford Cyrosystem Cobra low-temperature attachment.
Geometry. All e.s.d.'s (except the e.s.d. in the dihedral angle between two l.s. planes) are estimated using the full covariance matrix. The cell e.s.d.'s are taken into account individually in the estimation of e.s.d.'s in distances, angles and torsion angles; correlations between e.s.d.'s in cell parameters are only used when they are defined by crystal symmetry. An approximate (isotropic) treatment of cell e.s.d.'s is used for estimating e.s.d.'s involving l.s. planes.
Refinement. Refinement of *F*^2^ against ALL reflections. The weighted *R*-factor *wR* and goodness of fit *S* are based on *F*^2^, conventional *R*-factors *R* are based on *F*, with *F* set to zero for negative *F*^2^. The threshold expression of *F*^2^ > σ(*F*^2^) is used only for calculating *R*-factors(gt) *etc*. and is not relevant to the choice of reflections for refinement. *R*-factors based on *F*^2^ are statistically about twice as large as those based on *F*, and *R*- factors based on ALL data will be even larger.

### Fractional atomic coordinates and isotropic or equivalent isotropic displacement parameters (Å^2^)

**Table:**

	*x*	*y*	*z*	*U*_iso_*/*U*_eq_	
Cl1	0.01004 (4)	0.17798 (4)	0.21608 (3)	0.02610 (12)	
O1	0.64039 (12)	−0.00053 (10)	0.20712 (6)	0.0164 (3)	
O2	0.73070 (12)	−0.10345 (11)	0.31975 (6)	0.0177 (3)	
N1	0.60775 (13)	−0.11487 (12)	0.10350 (7)	0.0145 (3)	
N2	0.53262 (14)	−0.20840 (13)	0.07833 (7)	0.0156 (3)	
N3	0.54617 (13)	−0.18017 (13)	0.44999 (7)	0.0156 (3)	
N4	0.65437 (14)	−0.14862 (13)	0.42469 (7)	0.0153 (3)	
C1	0.79797 (17)	−0.01062 (16)	0.08926 (9)	0.0185 (4)	
H1A	0.8241	−0.0179	0.1391	0.022*	
C2	0.87259 (17)	0.04719 (17)	0.04601 (10)	0.0215 (4)	
H2A	0.9493	0.0813	0.0665	0.026*	
C3	0.83517 (18)	0.05518 (16)	−0.02710 (10)	0.0217 (4)	
H3A	0.8863	0.0945	−0.0566	0.026*	
C4	0.72305 (18)	0.00560 (16)	−0.05690 (9)	0.0191 (4)	
H4A	0.6986	0.0094	−0.1070	0.023*	
C5	0.64626 (17)	−0.04955 (15)	−0.01398 (9)	0.0169 (4)	
H5A	0.5683	−0.0814	−0.0342	0.020*	
C6	0.68519 (16)	−0.05749 (15)	0.05896 (9)	0.0144 (3)	
C7	0.58670 (16)	−0.08704 (15)	0.17178 (8)	0.0138 (3)	
C8	0.49935 (15)	−0.17256 (14)	0.18995 (8)	0.0125 (3)	
C9	0.46835 (16)	−0.24398 (15)	0.13014 (8)	0.0139 (3)	
C10	0.43446 (15)	−0.17598 (15)	0.25589 (8)	0.0129 (3)	
H10A	0.3941	−0.2562	0.2553	0.015*	
C11	0.32806 (16)	−0.08614 (15)	0.24690 (8)	0.0138 (3)	
C12	0.34955 (17)	0.03621 (15)	0.25601 (9)	0.0165 (3)	
H12A	0.4319	0.0640	0.2696	0.020*	
C13	0.25287 (17)	0.11770 (16)	0.24563 (9)	0.0181 (4)	
H13A	0.2686	0.2007	0.2516	0.022*	
C14	0.13238 (17)	0.07611 (16)	0.22627 (9)	0.0176 (4)	
C15	0.10766 (17)	−0.04447 (17)	0.21678 (9)	0.0194 (4)	
H15A	0.0250	−0.0719	0.2036	0.023*	
C16	0.20633 (16)	−0.12484 (16)	0.22686 (9)	0.0166 (4)	
H16A	0.1904	−0.2077	0.2199	0.020*	
C17	0.51224 (16)	−0.16574 (14)	0.32860 (8)	0.0131 (3)	
C18	0.46196 (16)	−0.19106 (15)	0.39214 (9)	0.0149 (3)	
C19	0.63540 (16)	−0.13831 (15)	0.35195 (9)	0.0142 (3)	
C20	0.76611 (17)	−0.13465 (16)	0.47292 (9)	0.0166 (4)	
C21	0.87757 (18)	−0.17791 (17)	0.45591 (10)	0.0220 (4)	
H21A	0.8809	−0.2158	0.4113	0.026*	
C22	0.98413 (19)	−0.16538 (18)	0.50458 (11)	0.0272 (4)	
H22A	1.0609	−0.1946	0.4931	0.033*	
C23	0.9802 (2)	−0.11074 (19)	0.56995 (11)	0.0304 (5)	
H23A	1.0538	−0.1025	0.6030	0.037*	
C24	0.8685 (2)	−0.06834 (19)	0.58662 (10)	0.0282 (5)	
H24A	0.8654	−0.0314	0.6315	0.034*	
C25	0.76066 (18)	−0.07926 (17)	0.53833 (9)	0.0217 (4)	
H25A	0.6841	−0.0494	0.5497	0.026*	
C26	0.37668 (17)	−0.34251 (16)	0.11547 (9)	0.0195 (4)	
H26A	0.4083	−0.4019	0.0845	0.029*	
H26B	0.2979	−0.3103	0.0917	0.029*	
H26C	0.3635	−0.3803	0.1605	0.029*	
C27	0.33330 (17)	−0.23080 (17)	0.39909 (9)	0.0198 (4)	
H27A	0.3299	−0.2573	0.4481	0.030*	
H27B	0.3099	−0.2970	0.3661	0.030*	
H27C	0.2756	−0.1642	0.3876	0.030*	
H1N2	0.541 (2)	−0.2396 (19)	0.0380 (7)	0.037 (7)*	
H1O2	0.702 (2)	−0.066 (2)	0.2832 (9)	0.057 (9)*	

### Atomic displacement parameters (Å^2^)

**Table:**

	*U*^11^	*U*^22^	*U*^33^	*U*^12^	*U*^13^	*U*^23^
Cl1	0.0214 (2)	0.0300 (3)	0.0268 (2)	0.0090 (2)	0.00288 (18)	−0.0010 (2)
O1	0.0231 (7)	0.0152 (6)	0.0111 (6)	−0.0047 (5)	0.0031 (5)	−0.0009 (5)
O2	0.0190 (7)	0.0226 (7)	0.0120 (6)	−0.0015 (5)	0.0034 (5)	0.0031 (5)
N1	0.0187 (8)	0.0152 (7)	0.0100 (7)	−0.0037 (6)	0.0032 (6)	−0.0012 (5)
N2	0.0206 (8)	0.0166 (7)	0.0102 (7)	−0.0027 (6)	0.0042 (6)	−0.0034 (5)
N3	0.0179 (7)	0.0178 (7)	0.0119 (7)	0.0001 (6)	0.0049 (5)	0.0015 (6)
N4	0.0181 (8)	0.0180 (7)	0.0099 (7)	−0.0006 (6)	0.0019 (6)	0.0016 (5)
C1	0.0184 (9)	0.0245 (9)	0.0127 (8)	0.0018 (7)	0.0027 (7)	0.0006 (7)
C2	0.0183 (9)	0.0264 (10)	0.0208 (9)	−0.0015 (8)	0.0062 (7)	−0.0010 (8)
C3	0.0250 (10)	0.0218 (9)	0.0210 (9)	0.0004 (8)	0.0124 (8)	0.0024 (7)
C4	0.0253 (10)	0.0216 (9)	0.0114 (8)	0.0039 (8)	0.0062 (7)	0.0028 (7)
C5	0.0178 (9)	0.0191 (9)	0.0136 (8)	0.0014 (7)	0.0021 (7)	−0.0002 (7)
C6	0.0166 (9)	0.0163 (8)	0.0114 (8)	0.0015 (7)	0.0057 (6)	0.0015 (6)
C7	0.0176 (9)	0.0149 (8)	0.0093 (7)	0.0035 (7)	0.0026 (6)	0.0011 (6)
C8	0.0149 (8)	0.0135 (7)	0.0091 (7)	0.0009 (7)	0.0020 (6)	0.0006 (6)
C9	0.0162 (8)	0.0148 (8)	0.0109 (7)	0.0020 (7)	0.0027 (6)	0.0017 (6)
C10	0.0159 (8)	0.0126 (8)	0.0106 (7)	−0.0022 (7)	0.0033 (6)	0.0007 (6)
C11	0.0172 (9)	0.0178 (8)	0.0070 (7)	0.0003 (7)	0.0039 (6)	0.0009 (6)
C12	0.0165 (9)	0.0179 (8)	0.0150 (8)	−0.0022 (7)	0.0018 (7)	0.0000 (7)
C13	0.0210 (9)	0.0168 (8)	0.0172 (8)	0.0003 (7)	0.0046 (7)	−0.0003 (7)
C14	0.0175 (9)	0.0224 (9)	0.0132 (8)	0.0051 (7)	0.0033 (7)	0.0010 (7)
C15	0.0143 (9)	0.0251 (9)	0.0189 (9)	−0.0019 (7)	0.0026 (7)	0.0000 (7)
C16	0.0195 (9)	0.0174 (8)	0.0133 (8)	−0.0026 (7)	0.0037 (7)	−0.0001 (6)
C17	0.0173 (8)	0.0133 (8)	0.0092 (7)	0.0008 (7)	0.0035 (6)	0.0007 (6)
C18	0.0190 (9)	0.0142 (8)	0.0116 (7)	0.0005 (7)	0.0030 (6)	0.0000 (6)
C19	0.0199 (9)	0.0129 (8)	0.0106 (7)	0.0012 (7)	0.0043 (6)	0.0003 (6)
C20	0.0201 (9)	0.0174 (8)	0.0114 (8)	−0.0011 (7)	−0.0008 (7)	0.0025 (6)
C21	0.0244 (10)	0.0213 (9)	0.0197 (9)	0.0043 (8)	0.0013 (7)	−0.0014 (7)
C22	0.0247 (10)	0.0277 (10)	0.0280 (10)	0.0033 (9)	−0.0003 (8)	0.0049 (8)
C23	0.0280 (11)	0.0364 (12)	0.0237 (10)	−0.0040 (9)	−0.0078 (8)	0.0058 (9)
C24	0.0329 (12)	0.0381 (12)	0.0125 (9)	−0.0084 (10)	−0.0008 (8)	−0.0003 (8)
C25	0.0244 (10)	0.0256 (10)	0.0154 (9)	−0.0008 (8)	0.0043 (7)	0.0005 (7)
C26	0.0232 (10)	0.0211 (9)	0.0140 (8)	−0.0055 (8)	0.0021 (7)	−0.0014 (7)
C27	0.0220 (10)	0.0260 (9)	0.0120 (8)	−0.0021 (8)	0.0046 (7)	0.0036 (7)

### Geometric parameters (Å, °)

**Table:**

Cl1—C14	1.7437 (18)	C11—C16	1.395 (2)
O1—C7	1.273 (2)	C11—C12	1.397 (2)
O2—C19	1.333 (2)	C12—C13	1.386 (2)
O2—H1O2	0.832 (10)	C12—H12A	0.95
N1—N2	1.3732 (19)	C13—C14	1.392 (2)
N1—C7	1.380 (2)	C13—H13A	0.95
N1—C6	1.426 (2)	C14—C15	1.384 (3)
N2—C9	1.342 (2)	C15—C16	1.394 (2)
N2—H1N2	0.856 (9)	C15—H15A	0.95
N3—C18	1.333 (2)	C16—H16A	0.95
N3—N4	1.3775 (19)	C17—C19	1.386 (2)
N4—C19	1.369 (2)	C17—C18	1.418 (2)
N4—C20	1.424 (2)	C18—C27	1.492 (2)
C1—C6	1.384 (2)	C20—C21	1.385 (3)
C1—C2	1.391 (2)	C20—C25	1.394 (2)
C1—H1A	0.95	C21—C22	1.384 (3)
C2—C3	1.391 (3)	C21—H21A	0.95
C2—H2A	0.95	C22—C23	1.387 (3)
C3—C4	1.387 (3)	C22—H22A	0.95
C3—H3A	0.95	C23—C24	1.382 (3)
C4—C5	1.389 (2)	C23—H23A	0.95
C4—H4A	0.95	C24—C25	1.389 (3)
C5—C6	1.390 (2)	C24—H24A	0.95
C5—H5A	0.95	C25—H25A	0.95
C7—C8	1.425 (2)	C26—H26A	0.98
C8—C9	1.389 (2)	C26—H26B	0.98
C8—C10	1.518 (2)	C26—H26C	0.98
C9—C26	1.488 (2)	C27—H27A	0.98
C10—C17	1.517 (2)	C27—H27B	0.98
C10—C11	1.526 (2)	C27—H27C	0.98
C10—H10A	1.00		
			
C19—O2—H1O2	107.5 (19)	C12—C13—H13A	120.5
N2—N1—C7	109.22 (13)	C14—C13—H13A	120.5
N2—N1—C6	120.74 (13)	C15—C14—C13	121.32 (16)
C7—N1—C6	129.89 (14)	C15—C14—Cl1	119.53 (14)
C9—N2—N1	108.57 (13)	C13—C14—Cl1	119.13 (14)
C9—N2—H1N2	131.3 (15)	C14—C15—C16	118.73 (17)
N1—N2—H1N2	119.4 (15)	C14—C15—H15A	120.6
C18—N3—N4	105.11 (13)	C16—C15—H15A	120.6
C19—N4—N3	110.79 (14)	C15—C16—C11	121.36 (16)
C19—N4—C20	129.18 (15)	C15—C16—H16A	119.3
N3—N4—C20	120.00 (13)	C11—C16—H16A	119.3
C6—C1—C2	119.28 (16)	C19—C17—C18	104.10 (14)
C6—C1—H1A	120.4	C19—C17—C10	134.34 (15)
C2—C1—H1A	120.4	C18—C17—C10	121.51 (15)
C3—C2—C1	120.14 (17)	N3—C18—C17	112.15 (15)
C3—C2—H2A	119.9	N3—C18—C27	120.06 (15)
C1—C2—H2A	119.9	C17—C18—C27	127.74 (15)
C4—C3—C2	119.90 (16)	O2—C19—N4	117.85 (15)
C4—C3—H3A	120.1	O2—C19—C17	134.30 (15)
C2—C3—H3A	120.1	N4—C19—C17	107.82 (14)
C3—C4—C5	120.44 (16)	C21—C20—C25	120.61 (17)
C3—C4—H4A	119.8	C21—C20—N4	120.64 (16)
C5—C4—H4A	119.8	C25—C20—N4	118.73 (16)
C4—C5—C6	119.01 (17)	C22—C21—C20	119.29 (18)
C4—C5—H5A	120.5	C22—C21—H21A	120.4
C6—C5—H5A	120.5	C20—C21—H21A	120.4
C1—C6—C5	121.18 (16)	C21—C22—C23	120.80 (19)
C1—C6—N1	119.26 (15)	C21—C22—H22A	119.6
C5—C6—N1	119.56 (15)	C23—C22—H22A	119.6
O1—C7—N1	122.74 (15)	C24—C23—C22	119.54 (19)
O1—C7—C8	131.14 (15)	C24—C23—H23A	120.2
N1—C7—C8	106.11 (14)	C22—C23—H23A	120.2
C9—C8—C7	106.55 (14)	C23—C24—C25	120.58 (19)
C9—C8—C10	124.61 (15)	C23—C24—H24A	119.7
C7—C8—C10	128.14 (14)	C25—C24—H24A	119.7
N2—C9—C8	109.43 (15)	C24—C25—C20	119.19 (18)
N2—C9—C26	119.19 (15)	C24—C25—H25A	120.4
C8—C9—C26	131.30 (15)	C20—C25—H25A	120.4
C17—C10—C8	118.70 (14)	C9—C26—H26A	109.5
C17—C10—C11	111.73 (13)	C9—C26—H26B	109.5
C8—C10—C11	108.91 (13)	H26A—C26—H26B	109.5
C17—C10—H10A	105.5	C9—C26—H26C	109.5
C8—C10—H10A	105.5	H26A—C26—H26C	109.5
C11—C10—H10A	105.5	H26B—C26—H26C	109.5
C16—C11—C12	118.34 (16)	C18—C27—H27A	109.5
C16—C11—C10	120.15 (15)	C18—C27—H27B	109.5
C12—C11—C10	121.48 (15)	H27A—C27—H27B	109.5
C13—C12—C11	121.21 (17)	C18—C27—H27C	109.5
C13—C12—H12A	119.4	H27A—C27—H27C	109.5
C11—C12—H12A	119.4	H27B—C27—H27C	109.5
C12—C13—C14	119.04 (16)		
			
C7—N1—N2—C9	−2.66 (18)	C10—C11—C12—C13	178.00 (15)
C6—N1—N2—C9	−178.76 (15)	C11—C12—C13—C14	0.5 (3)
C18—N3—N4—C19	1.09 (18)	C12—C13—C14—C15	−0.5 (3)
C18—N3—N4—C20	−177.23 (15)	C12—C13—C14—Cl1	177.88 (13)
C6—C1—C2—C3	−1.5 (3)	C13—C14—C15—C16	−0.1 (3)
C1—C2—C3—C4	0.1 (3)	Cl1—C14—C15—C16	−178.44 (13)
C2—C3—C4—C5	1.6 (3)	C14—C15—C16—C11	0.7 (3)
C3—C4—C5—C6	−1.9 (3)	C12—C11—C16—C15	−0.7 (2)
C2—C1—C6—C5	1.1 (3)	C10—C11—C16—C15	−178.61 (15)
C2—C1—C6—N1	−178.90 (16)	C8—C10—C17—C19	9.7 (3)
C4—C5—C6—C1	0.6 (3)	C11—C10—C17—C19	−118.3 (2)
C4—C5—C6—N1	−179.41 (15)	C8—C10—C17—C18	−167.66 (15)
N2—N1—C6—C1	−147.80 (16)	C11—C10—C17—C18	64.3 (2)
C7—N1—C6—C1	37.0 (3)	N4—N3—C18—C17	−0.72 (19)
N2—N1—C6—C5	32.2 (2)	N4—N3—C18—C27	176.97 (15)
C7—N1—C6—C5	−142.99 (18)	C19—C17—C18—N3	0.11 (19)
N2—N1—C7—O1	−177.20 (15)	C10—C17—C18—N3	178.15 (15)
C6—N1—C7—O1	−1.6 (3)	C19—C17—C18—C27	−177.37 (17)
N2—N1—C7—C8	3.27 (18)	C10—C17—C18—C27	0.7 (3)
C6—N1—C7—C8	178.90 (16)	N3—N4—C19—O2	177.35 (14)
O1—C7—C8—C9	177.87 (17)	C20—N4—C19—O2	−4.5 (3)
N1—C7—C8—C9	−2.66 (18)	N3—N4—C19—C17	−1.05 (19)
O1—C7—C8—C10	7.1 (3)	C20—N4—C19—C17	177.07 (16)
N1—C7—C8—C10	−173.38 (15)	C18—C17—C19—O2	−177.46 (18)
N1—N2—C9—C8	0.91 (19)	C10—C17—C19—O2	4.9 (3)
N1—N2—C9—C26	178.13 (15)	C18—C17—C19—N4	0.56 (18)
C7—C8—C9—N2	1.11 (19)	C10—C17—C19—N4	−177.10 (17)
C10—C8—C9—N2	172.25 (15)	C19—N4—C20—C21	−39.9 (3)
C7—C8—C9—C26	−175.66 (17)	N3—N4—C20—C21	138.11 (17)
C10—C8—C9—C26	−4.5 (3)	C19—N4—C20—C25	141.73 (18)
C9—C8—C10—C17	139.90 (16)	N3—N4—C20—C25	−40.3 (2)
C7—C8—C10—C17	−50.9 (2)	C25—C20—C21—C22	−0.2 (3)
C9—C8—C10—C11	−90.77 (19)	N4—C20—C21—C22	−178.54 (17)
C7—C8—C10—C11	78.4 (2)	C20—C21—C22—C23	0.3 (3)
C17—C10—C11—C16	−126.08 (16)	C21—C22—C23—C24	0.0 (3)
C8—C10—C11—C16	100.84 (17)	C22—C23—C24—C25	−0.5 (3)
C17—C10—C11—C12	56.1 (2)	C23—C24—C25—C20	0.6 (3)
C8—C10—C11—C12	−77.01 (19)	C21—C20—C25—C24	−0.3 (3)
C16—C11—C12—C13	0.1 (2)	N4—C20—C25—C24	178.12 (17)

### Hydrogen-bond geometry (Å, °)

**Table:**

*D*—H···*A*	*D*—H	H···*A*	*D*···*A*	*D*—H···*A*
O2—H1O2···O1	0.83 (1)	1.67 (1)	2.5025 (17)	176 (3)
N2—H1N2···N3^i^	0.86 (1)	1.90 (1)	2.7544 (19)	174 (2)
C13—H13A···O2^ii^	0.95	2.58	3.375 (2)	142
C4—H4A···Cg1^iii^	0.95	2.67	3.5088 (18)	147
C24—H24A···Cg1^iv^	0.95	2.86	3.745 (2)	155

Symmetry codes: (i) *x*, −*y*−1/2, *z*−1/2; (ii) −*x*+1, *y*+1/2, −*z*+1/2; (iii) −*x*+1, −*y*, −*z*; (iv) −*x*+1, −*y*, −*z*+1.
